# A dose-effect relationship for deltaretrovirus-dependent leukemogenesis in sheep

**DOI:** 10.1186/1742-4690-6-30

**Published:** 2009-04-03

**Authors:** Carole Pomier, Maria Teresa Sanchez Alcaraz, Christophe Debacq, Agnes Lançon, Pierre Kerkhofs, Lucas Willems, Eric Wattel, Franck Mortreux

**Affiliations:** 1 CNRS FRE-3011 - Université Lyon I, Oncovirologie et Biothérapies, Centre Léon Bérard, Lyon, France; 2 FUSAGx, Molecular and cellular biology, Gembloux, Belgium; 3 Hôpital Edouard Herriot, Service d'Hématologie, Pavillon E, Lyon, France; 4 Veterinary and Agrochemical Research Centre, Department of Virology, Uccle, Belgium

## Abstract

**Background:**

Retrovirus-induced tumors develop in a broad range of frequencies and after extremely variable periods of time, from only a few days to several decades, depending mainly on virus type. For hitherto unexplained reasons, deltaretroviruses cause hematological malignancies only in a minority of naturally infected organisms and after a very prolonged period of clinical latency.

**Results:**

Here we demonstrate that the development of malignancies in sheep experimentally infected with the deltaretrovirus bovine leukemia virus (BLV) depends only on the level of BLV replication. Animals were experimentally infected with leukemogenic or attenuated, but infectious, BLV molecular clones and monitored prospectively through 8 months for viral replication. As early as 2 weeks after infection and subsequently at any time during follow-up, leukemogenic viruses produced significantly higher absolute levels of reverse transcription (RT), clonal expansion of infected cells, and circulating proviruses with RT- and somatic-dependent mutations than attenuated viruses. These differences were only quantitative, and both kinds of viruses triggered parallel temporal fluctuations of host lymphoid cells, viral loads, infected cell clonality and proliferation.

**Conclusion:**

Deltaretrovirus-associated leukemogenesis in sheep appears to be a two-hit process over time depending on the amounts of first horizontally and then vertically expanded viruses.

## Background

Oncogenic retroviruses are known to cause cancers by the acquisition and expression of host-derived oncogenes, by the insertional activation of host cell oncogenes, and the expression of viral proteins such as those encoded by the *tax *or the *HBZ *genes of human T-cell leukemia virus (HTLV) [1], or by the envelope gene of the Jaagsiekte sheep retrovirus (JSRV) [2,3]. Tumors develop in a very broad range of frequencies and after extremely variable periods of time, depending mainly on the virus type, but also on the viral strain, the host genetic background, exogenous cofactors, and combinations thereof. For example, JSRV can induce lung adenocarcinoma in newborn lambs in as little as 10 days following experimental inoculation [4]; while after infection with acute transforming retroviruses such as the Rous sarcoma virus (RSV) infection, tumors develop after a few weeks in almost all infected chickens [5]. In strong contrast, less than 5% of deltaretrovirus-infected organisms develop leukemia after very prolonged periods of latency. In fact, leukemogenic deltaretroviruses are the least oncogenic retroviruses in term of disease penetrance in their naturally infected host. In humans, adult T-cell leukemia/lymphoma (ATLL) occurs only in about 1–3% of individuals infected with HTLV-1[6]. In monkeys, the incidence of STLV-associated malignancies seems along the same order [7], while in infected cows BLV triggers leukemia in about 5% of the animals [8]. In addition to a low incidence, deltaretrovirus-associated malignancies commonly develop after a prolonged period of latency that encompasses about two thirds of the predicted lifespan of the respective hosts [6-8]. This very low leukemogenic effect *in vivo *is in sharp contrast with the extremely high level of oncogenicity that characterizes these viruses *ex vivo*. They encode proteins such as Tax or HBZ which can transform cells by interfering with numerous cellular pathways involved in tumor promotion and maintenance [1].

For HTLV-1, infection occurring early in the life of the host is crucial in the development of ATLL[6]. Furthermore, the disease has been found to occur more frequently and more rapidly in individuals suffering from strongyloidiasis or infectious dermatitis, two clinical conditions characterized at the asymptomatic phase of HTLV-1 infection, by extremely high circulating proviral loads [9-11]. Similarly, ATLL cases retrospectively analyzed at the asymptomatic phase of infection have been found to display higher circulating proviral loads than disease-free carriers from the same geographic area [12]. In animal models, such as squirrel monkeys infected with HTLV-1 or sheep infected with BLV, we and others previously observed that deltaretroviral leukemogenesis regularly includes a progressive increase of proviral load related to the proliferation of preleukemic clones [13,14].

Although they are retrospective or uncontrolled, these data led us to test the hypothesis of a link between intense deltaretroviral replication and subsequent tumor development. It is possible to genetically modulate the oncogenicity of BLV molecular clones without altering their ability to experimentally infect sheep [8,15,16]. We reasoned that the prospective comparison of early BLV replication in animals infected with leukemogenic versus attenuated infectious molecular clones would be appropriate for investigating the implication of qualitative and/or quantitative alterations of deltaretrovirus replication in the subsequent development of malignancies.

## Results

**Figure 1:**
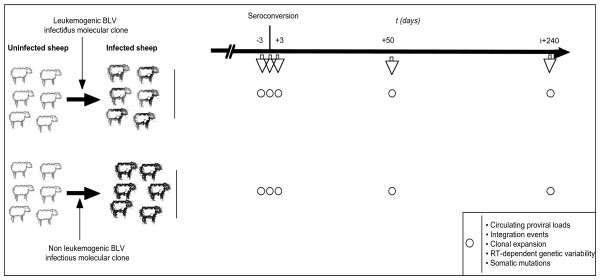
**Experimental strategy**. Twelve sheep were infected by direct inoculation of a cloned BLV provirus. Six animals (top) were infected with the leukemogenic BLV infectious molecular clones pBLV344, pBLVTax106+293 and pBLVA60V (2 animals per clone). These molecularly cloned viruses induce leukemias in almost all infected animals. Six additional sheep (bottom) were infected with the infectious, but attenuated, cloned viruses pBLVCRX3, pBLVIG4 and pBLVCRE3X [15] (2 animals per clone). All 12 experimentally infected sheep seroconverted and developed persistent infection. For each animal, blood samples were prospectively collected by jugular venipuncture and prepared for analyses at the date of seroconversion, 3 days before, 3 and 50 days after, and 240 days after experimental infection. All animals infected with leukemogenic viruses were investigated prior to tumor onset. The 2 groups of infected sheep were compared for BLV replication by using quantitative analyses of circulating proviral loads, reverse-transcription (RT) events, RT-dependent genetic variability, clonal expansion, and somatic mutations.

Twelve sheep were infected by direct inoculation of a cloned BLV provirus, as previously described [14]. Six animals were infected with the leukemogenic BLV infectious molecular clones pBLV344, pBLVTax106+293 and pBLVA60V (2 animals per clone, see methods). These molecularly cloned viruses are characterized as leukemogenic strains because they induce leukemias in almost all infected sheep after a relatively short clinical latency period ranging from 1 to 4 years. Six additional sheep were infected with cloned viruses pBLVCRX3, pBLVIG4 and pBLVCRE3X [15] (2 animals per clone, see methods). While mutated in different proviral sequences including R3, G4 or LTR, these BLV molecular clones remained infectious but have lost the ability to induce leukemia in sheep after a mean of 66 months follow-up (26 – 84 months). Two additional sheep inoculated with a BLV-negative solution served as controls. All 12 experimentally infected sheep seroconverted and developed persistent infection. The seroconversion occurred 24, 28, 42, 58, 65 and 79 (mean ± SD, 49.3 ± 18) days post-infection for the leukemogenic BLV infected sheep 4546, 4536, 4544, 1048, 4545 and 4535; and 21, 28, 31, 35, 49 and 65 (mean ± SD, 38.2 ± 12.5) days post-infection for the attenuated BLV infected sheep 4538, 4543, 4537, 4541, 4542 and 4539 respectively. This difference was not statistically significant (p > 0.05, Mann-Whitney test) indicating that attenuated BLV molecular clones did not induce delayed seroconversion. For each animal, blood samples were prospectively collected by jugular venipuncture and prepared for analyses on the date of seroconversion, 3 days before, and 3, 50, and 240 days after experimental infection. All animals infected with leukemogenic viruses were investigated prior to tumor onset. As previously described [17], experimental primary BLV infection resulted in transient hyperleukocytosis and B lymphocytosis without significant differences between animals infected with leukemogenic versus attenuated viruses. This indicates that initial infection with the two different groups of viruses resulted in similar primary symptoms. The 2 groups of infected sheep were compared for BLV replication using the experimental strategy illustrated in Figure 1.

**Figure 2:**
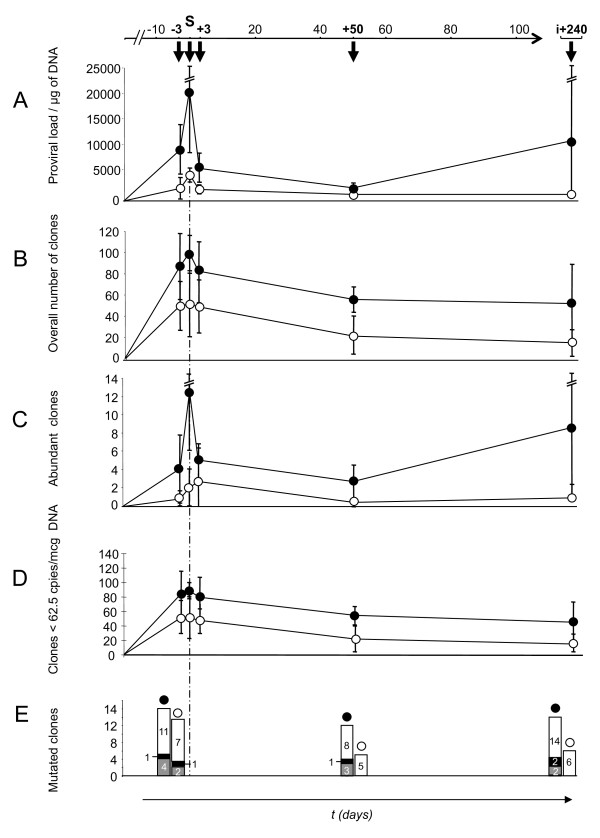
**Temporal fluctuations of leukemogenic versus attenuated virus replication *in vivo *after experimental infection**. Early Bovine Leukemia Virus replication was compared over time between sheep infected with attenuated (open circles) versus leukemogenic (black circles) infectious molecular clones. S-3: 3 days before serconversion, S: seroconversion, S+3: 3 days after seroconversion, S+50: 50 days after seroconversion, i+240: 240 days post-incoculation. Comparisons were made for circulating proviral loads (A), overall measured RT events (B), abundant clones (C), less extensively expanded clones (clones < 62.5 copies per 1 mcg of DNA) (D), somatic (black histograms) and RT-dependent (grey histograms) proviral mutations; white histograms represent unmutated clones (E). Error bars are ± standard error of mean.

**Figure 3:**
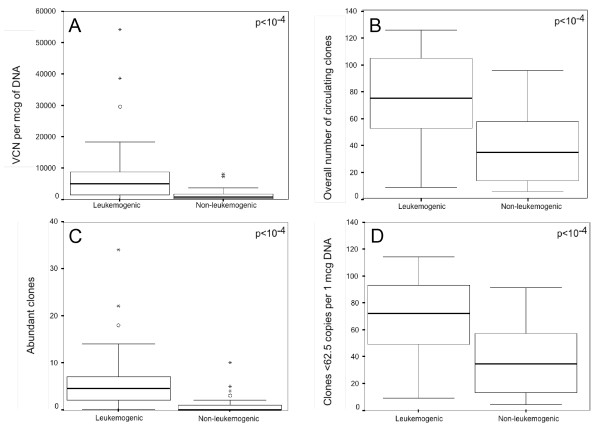
**Leukemogenic molecular clones trigger higher levels of early BLV replication than attenuated infectious molecular clones *in vivo***. Time-point data were pooled and compared between leukemogenic and attenuated infections. A: proviral copy (VCN) number per mcg of DNA; B: overall number of circulating infected cellular clones; C: Numbers of abundant clones were defined as those giving two or more signals after semiquantitative quadruplicate IPCR, i.e. those corresponding to ≥ 62.5 copies in 1 mcg of circulating DNA [14] ; D: infected cellular clones with < 62,5 copies per mcg of DNA. The boxes represent the difference between the 75th percentile and the 25th percentile of the variables (i.e., the interquartile range). Within the inset box, the median is represented by a thick horizontal line. Lines from the ends of the box extend as far as the most extreme values not considered outliers. Points more than 1.5 times the interquartile range from the ends of the box are labeled as outliers ([open circle]) or as extreme values ([low asterisk]).

We first evaluated the effect of oncogenic versus attenuated viruses on the overall amount of proviral sequences over time. Quantitative real-time PCR amplification of the conserved BLV *tax *gene was carried out in 60 samples isolated from the 12 experimentally infected sheep during the first 8 months of the infection (Figure 2A). The mean BLV copy numbers per mcg of DNA in the 30 samples derived from the 6 animals infected with leukemogenic infectious molecular clones was ~6 times higher than that of animals experimentally infected with attenuated strains (8893 vs 1483, p < 10^-4^, Mann-Whitney test) (Figure 3A). Figure 2A shows an increase in the mean proviral load in leukemogenic BLV carriers at 240 days postinfection. However this effect was related to an extreme proviral load, up to 54000 proviral copies per mcg of DNA, carried by the sheep 4535 (not shown). Except for this time-point, the fluctuations in the circulating proviral loads of leukemogenic viruses paralleled those of attenuated strains. Altogether these data indicated that a simple quantitative effect distinguishes the circulating proviral loads of leukemogenic from those of attenuated viruses without modifying their qualitative distribution.

**Figure 4:**
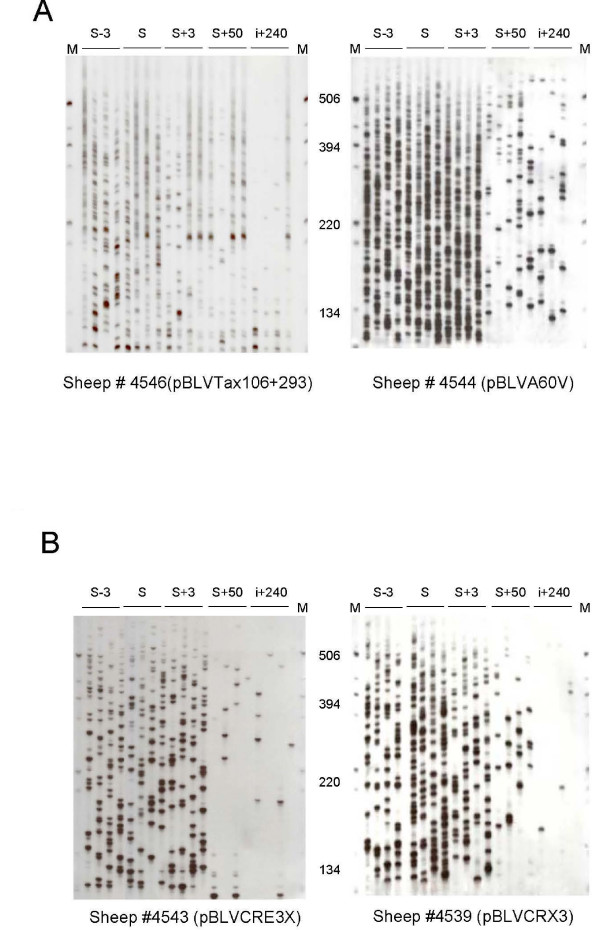
**Clonal distribution of BLV positive cells in sheep infected by leukemogenic versus attenuated viruses**. Autoradiogram of run-off products (see method). The degree of clonal expansion of infected cells over time was measured by semiquantitative quadruplicate IPCR for each animal. (A) Animals #4546 and #4544 were inoculated with the leukemogenic molecular clones pBLVTax106+293 and pBLVA60V, respectively. (B) Animals #4543 and #4539 were inoculated with the attenuated molecular clones pBLVCRE3X and pBLVCRX3, respectively.

As with other deltaretroviruses, BLV possesses two routes of replication that include reverse-transcription and the clonal expansion of its host cells [14]. In a recent report, we have shown that both co-exist during primary infection while clonal expansion seems to predominate during the chronic phase of the infection [18]. As leukemogenic molecular clones triggered elevated circulating proviral loads *in vivo*, we next investigated whether these differences in circulating proviral loads depended on the level of RT-based, horizontal BLV replication or rather on the level of clonal expansion of BLV positive cells. As both processes co-exist during the early phase of the infection [18,19], we focused our analyses on the first months following experimental infection. Inverse PCR amplification of BLV 3' integration sites was carried out as already described [14,18] for estimating the number of BLV integration events (i.e. the number of entire horizontal RT-dependent transmission events that resulted in proviral integration and clonal expansion of infected cells during primary infection). The number of BLV integration events was measured over time in each animal at the same times that the proviral loads were quantified (Figures 2B and 4). After quadruplicate IPCR, the mean number of integration events detected in the 30 samples deriving from the 6 animals infected with leukemogenic infectious molecular clones varied from 9 to 126; those data from animals infected with attenuated infectious strains varied from 6 to 96 (Figure 3B). The mean ± standard error of mean (s.e.m.) and the median of detected integration events were 76 ± 6 and 75 for samples derived from animals infected with leukemogenic clones, and 39 ± 5 and 35 for samples derived from animals infected with attenuated clones. As shown in figure 3, the difference between the two groups of samples was statistically significant (p < 10^-4^, Mann-Whitney test). In contrast, temporal fluctuations of BLV integration events were found to be parallel for leukemogenic and attenuated infectious molecular clones (Figure 2B). These data indicated that BLV leukemogenicity depends on the level of RT-dependent BLV replication *in vivo*.

Next, we tested whether the leukemogenicity of molecularly cloned viruses impacted the level of cell-associated BLV replication. To this end we measured by semiquantitative quadruplicate IPCR the degree of clonal expansion of infected cells over time in each animal (Figures 2C, 2D and 4). The mean number of abundant clones (detected ≥ 2/4 IPCR experiments corresponding to ≥ 62.5 copies per 1 μg of DNA [14]) in the 30 samples derived from the 6 animals infected with leukemogenic infectious molecular clones varied from 0 in 2 samples to 34; in animals infected with attenuated infectious strains, the numbers varied from 0 in 16 samples to 10 (Figure 2C). The mean ± standard error of mean (s.e.m.) and median of the number of circulating abundant BLV-positive clones were 6.5 ± 1.3 and 4.5 for samples derived from animals infected with leukemogenic clones, and 1.2 ± 0.4 and 0 for samples derived from animals infected with leukemogenic clones. As shown in figure 3C, the difference between the two groups was statistically significant (p < 10^-4^, Mann-Whitney test). As described above for proviral load, the number of abundant infected clones was atypically extremely high (n = 34) in the sheep 4535 at the point i+240, thereby increasing the mean value for the leukemogenic BLV infected group. Except for this time-point, the degree of clonal expansion was roughly parallel between animals infected with leukemogenic versus attenuated BLV infectious molecular clones (Figures 2C, 2D and 4), indicating that attenuated viruses do not alter the dynamic of the infection over time. Altogether these results demonstrate that BLV leukemogenicity depends on the degree of vertical transmission of BLV via infected cell clonal expansion.

Genetic variability of retroviruses goes hand in hand with the degree of retroviral replication. In contrast with other retroviruses, members of HTLV-BLV genus involve two distinct routes of genetic variability *in vivo *including RT- and somatic-associated mechanisms [14,18-20]. The cycles of reverse transcription govern RT-dependent genetic variability, whereas extended clonal expansion is accompanied by somatic rearrangements of integrated proviruses [19,20]. Having identified high levels of both BLV replication mechanisms with leukemogenic infectious proviruses, we investigated whether the number of RT- and somatic-dependent substitutions tended to be higher in animals infected with leukemogenic BLV molecular clones. To this end, IPCR products of 12 blood samples, harvested 3 days before, 50 days after seroconversion and 240 days post infection of 4 sheep (2 from each infection category) were cloned and sequenced. As previously described, the alignment of sequenced IPCR products allowed us to clearly distinguish provirus mutations originating from somatic- or RT-dependent rearrangements [14,18-20]. Overall, 834 sequenced molecular clones were obtained and the alignment of the 3'LTR sequences with respect to their integration sites allowed the identification of 67 distinct infected cellular clones. Their overtime distributions were 26, 17 and 24 infected cellular clones at 3 days before, 50 days after seroconversion and 240 days post-inoculation, respectively. The figure 2E shows the distribution of the mutated cellular clones during the course of infection with leukemogenic or attenuated BLV infectious proviruses. For the leukemogenic-BLV infected sheep, sequence alignments allowed the detection of 4/16, 3/12 and 2/18 mutated cellular clones with RT-associated mutation in their proviral sequences at 3 days before, 50 days after seroconversion and 240 days post-inoculation, respectively. In comparison, at these respective time-points, attenuated infections displayed 2/10, 0/5 and 0/6 cellular clones with RT-associated mutation in proviruses. Although different, these values only reflected the higher amount of clones generated after infection with leukemogenic viruses: 9 substitutions in 46 cellular clones versus 2 in 21 cellular clones, respectively. As shown in figure 2E, similar results were obtained when analyzing the somatic mutation frequencies. Despite an increased number of cellular clones harboring somatic mutated proviruses in the leukemogenic BLV infection, the difference was not significant when the number of detected clones and the amount of sequenced DNA were taken into account for leukemogenic versus attenuated viruses: 4 substitutions in 46 cellular clones (484 sequenced molecular clones, 203 kb of data) versus 1 in 21 cellular clones (350 sequenced molecular clones, 147 kb of data), respectively (Figure 2E). These data indicate that leukemogenic BLV infection, in comparison to infection with attenuated BLV infectious molecular clones, could be characterized by a higher amount of infected cells with mutated proviruses resulting from RT- and/or somatic-dependent events. However, the frequencies of such mutated clones did not change significantly between the two categories of viruses.

## Discussion

This work is the first prospective controlled investigation of a deltaretrovirus-dependent leukemogenesis that takes into account the two time-dependent routes of deltaretroviruses replication [14,19,20]. The data show that, as early as 2 weeks after infection and subsequently at any time during follow-up, leukemogenic viruses displayed significantly higher levels of RT and absolute number of RT-dependent mutation loads, clonal expansion, and clonal expansion-dependent somatic mutation loads than attenuated viruses.

The attenuated viruses correspond to mutated proviral sequences that retain infectious capacities while displaying reduced oncogenic clinical effect after experimental infection. To date, no BLV-related malignancy has been described in animals experimentally infected with the mutants used in the present study. Other attenuated BLV proviruses, such as those impaired in the accessory proteins R3 and G4 or transmembrane protein TM can induce lymphoma but at extremely low frequency (≤ 5%) and after a prolonged period of latency [21]. These molecular tools rendered it possible to prospectively compare the replication pattern of leukemogenic versus attenuated BLV strain *in vivo*.

Decreased circulating proviral loads have been recently evidenced at 7 months after experimental infection of sheep with BLV strains carrying mutations in the genes that encode R3 and G4 accessory proteins [21]. The present results confirm and extend these data with other BLV mutants and reveal that the two routes of BLV replication (i.e. reverse transcription and clonal expansion) distinguish the two kinds of viruses, and therefore are both involved in BLV-dependent leukemogenesis in sheep. Furthermore, by analyzing BLV replication over time, our study demonstrates that the differences in the replicative pattern between leukemogenic and attenuated strains are only quantitative and that both kinds of viruses trigger parallel temporal fluctuations of viral loads, infected cell clonality, and proliferation.

Sheep were experimentally infected by intradermal injection of plasmid DNA, which is not the natural route of infection for BLV. The virus is transmitted horizontally through the transfer of infected cells via direct contact, through milk and blood. The route of infection may influence the subsequent cellular immune response and thereby the replication level. This has been documented with HTLV-1 in experimentally infected adult rats, which display significantly lower levels of cellular immune response after gastrointestinal exposure than after intravenous or intraperitoneal inoculation [22]. Therefore, it will be interesting to test whether the replication and pathogenicity of leukemogenic or attenuated BLV proviruses could be influenced by the route of infection.

Deltaretovirus-associated cancers are diseases of long latency, and the present results suggest that, in addition to the duration of the infection, a dose effect governs BLV associated leukemogenesis. Leukemogenic viruses display an initial burst of intense horizontal RT-dependent replication that is followed by the extensive clonal expansion of infected cells. This expansion results in a disease penetrance rate of ~100% coupled with an extremely short period of clinical latency in sheep. In contrast, the attenuated virus replicates poorly by RT and clonal expansion. It either leaves animals free of tumors or causes disease in a minority of infected animals only after a prolonged period of latency. From these data, it is possible to propose that infection duration and viral dose, which depends on both RT and clonal expansion, are the two key parameters for tumor formation. In this model, the lower the replicative viral rate, the longer the infection course needed for triggering tumors. Knowing that for sheep infected with wild type BLV proviruses, premalignant clones are detected as early as 15 days from experimental infection [14]; the present results suggest that the burst of RT-dependent BLV replication that characterizes early infection helps to generate premalignant cells whose subsequent extensive clonal expansion contributes to transform.

In conclusion these results validate the use of circulating proviral loads as a clinical prognostic tool for managing deltaretroviral infections and suggest that targeting both RT and clonal expansion would be of interest for preventing associated malignancies.

## Methods

### Experimental BLV infection of sheep

Fourteen one-year-old sheep were kept under controlled conditions at the Veterinary and Agrochemical Research Centre (Machelen, Belgium). The handling of animals and the experimental procedures were approved by the ethics committee and were conducted in accordance with institutional and national guidelines for animal care and use. Twelve sheep were experimentally infected with BLV infectious molecular clones as previously described [17]. Briefly, 100 μg of circular plasmid DNA was mixed with 200 μg of Dotap (Roche Diagnostics), and the mixture was injected intradermally into the back of the sheep. Six animals were infected with the leukemogenic BLV infectious molecular clones pBLV344, pBLVTax106+293 and pBLVA60V (2 animals per clone). The pBLV344 was the wild type infectious molecular clone. The pBLVTax106+293 was mutated in the Tax phosphorylation sites[23], and pBLVA60V was mutated at the codon 60 of the GP30 transmembrane gene from alanine into valine [24]. Two of the 6 animals, sheep #4535 and #4536, infected with pBLV344 were previously analyzed for BLV replication and genetic variability [18]. Six additional sheep were infected with cloned viruses pBLVCRE3X, pBLVCRX3 and pBLVIG4 [15] (2 animals per clone). The pBLVCRE3X was mutated for CRE imperfect sequences to TGACGTCA [25] whereas pBLVCRX3 was deleted in R3 [8] and pBLVIG4 harbored a stop codon in the G4 open reading frame[16]. Two of the 6 animals infected with those attenuated infectious viruses, sheep #4537 and #4538, infected with pBLVIG4 were previously analyzed for BLV replication and genetic variability [18]. Two additional sheep, #4533 and 4534, inoculated with a BLV-negative Dotap solution served as uninfected controls. Twice a week, the total leukocyte counts were determined by using a Coulter counter ZN, and the number of lymphocytes was estimated after examination under the microscope after staining with May-Grunwald Giemsa. In parallel, the sera from each sheep were analyzed for BLV seropositivity as described [26].

### Measurement of circulating BLV proviral load

The circulating amounts of BLV proviral sequences were measured by LightCycler quantitative PCR as described [14]. Briefly, the reaction mixture included polymerase (LightCycler Kit Fast Start DNA Master Hybridization Probes; Roche), 2 mM MgCl 2, 500 nM primer BLVQF, 500 nM primer BLVQR, both targeting the Px region, and 100 nM donor probe 3' end labeled with fluorescein and 200 nM acceptor probe 5' end labeled with LC Red640. Standardization of the amount of DNA subjected to quantification was performed with the sheep beta-globin gene as an internal standard [27].

### Detection and quantification of the clonal distribution of circulating BLV positive cells in vivo

BLV integration was analyzed by Inverse Polymerase Chain Reaction (IPCR) as described [14]. Briefly, two micrograms of DNA were digested by 20 U NlaIII and 20 units of MfeI in 1× NlaIII-MfeI buffer for 3 hours at 37°C. MfeI digestion was performed in order to avoid the amplification of a 536 bp segment of the 5' LTR complementary to the set of 3' IPCR primers. One microgram of digested DNA was circularized for 16 hours at 16°C with 20 U of T4 DNA ligase. Samples were analyzed in quadruplicate, as previously described for BLV and HTLV-1 [14,28]: 4 × 500 ng of circularized DNA were amplified for 39 cycles using 200 μM of the primer pair BLV3'S and BLV3'AS. Amplifications were performed using 3.5 units of the Pfu DNA polymerase with thermal cycling parameters as follows: 95°C 10 minute, 35 × (95°C 1 min, 60°C 1 minute, 72°C 3 minutes), and a final elongation step of 10 minutes at 72°C. The length polymorphism analysis of 3' BLV flanking sequences was performed by making a run-off. This method consists in the linear PCR amplification of the provirus 3' extremities together with their flanking sequences. Two microliters of amplified IPCR products were submitted to 10 cycles of linear PCR with 2 μM of 5'-^32^P-radiolabeled primer BLV3'RO. Run-off products were analyzed on a 6% sequencing gel. As previously described [14], the stochastic nature of BLV IPCR was found to appear at BLV integration site frequencies ranging between 25 and 2400 copies of the BLV provirus per mcg of blood DNA. At copy numbers ranging from 1200 to 2400, 62.5 to 1200, and 25 to 62.5, detection was 3/4, 2/4, and 1/4, respectively. Accordingly, DNA samples from BLV infected animals were analyzed in quadruplicate (4 × 0.5 mcg).

### Assessment of BLV genetic variability in vivo

The cloning and sequencing of 3'LTR-integration site PCR fragments were performed as previously described [14]. Briefly, purified IPCR products were ligated with SmaI-digested and M13mp18 replicative form DNA. After transformation of Escherichia coli XL1, recombinant M13 plaques were screened by hybridization with the BLV3'RO oligonucleotides. Single-stranded templates were sequenced using fluorescent dideoxynucleotides. The sequenced products were resolved on an Applied Biosystems 377A DNA sequencer with 377A software. Sequence alignments were performed with Sequence Navigator Software.

### Statistical analysis

SPSS statistical software version 11 was used for analyses. The correlation of data was assessed by Spearman's *Rho *nonparametric method. P < 0.05 was considered significant in all analyses.

## Competing interests

The authors declare that they have no competing interests.

## Authors' contributions

CP carried out the most experimental work. MTSA, CD and FM performed the sample collections. AL, CP and FM performed the sequencing of IPCR products and the determination of the proviral loads. PK and LW were responsible for the sheep studies and participated in the interpretation of data. FM and EW were responsible for the design of the study and its coordination. CP, EW, and FM wrote the manuscript. All authors read and approved the final manuscript.
